# Pressure ulcers in individuals with spinal cord injury: a comparative analysis of healing outcomes in inpatient and outpatient settings

**DOI:** 10.1038/s41393-026-01267-1

**Published:** 2026-09-07

**Authors:** Maria Rasenack, Sabrina Imhof, Patrick Klein, Patrick Freund, Björn Zörner

**Affiliations:** 1 Spinal Cord Injury Centre, Balgrist University Hospital, Zurich, Switzerland; 2 Swiss Paraplegic Centre and Swiss Paraplegic Research, Nottwil, Switzerland

**Keywords:** Spinal cord diseases, Skin manifestations

## Abstract

**Background:**

Pressure ulcers (PUs) are a common and serious secondary complication in individuals with spinal cord injury (SCI). Effective treatment strategies are crucial for improving patient outcomes. However, it remains unclear whether inpatient or outpatient treatment leads to better healing outcomes.

**Study design:**

Retrospective cohort study.

**Objective:**

This study aimed to compare the healing progression of PUs grade 2 in SCI patients in the inpatient versus outpatient setting.

**Setting:**

Spinal Cord Injury Centre, Balgrist University Hospital, Zurich, Switzerland.

**Methods:**

Data from SCI patients treated for PUs between July 2017 and July 2019 were analysed retrospectively. Wound characteristics, localization, and healing progression were recorded for PUs grade 2. The primary outcome was the time to complete wound healing, analysed by localization and treatment setting. Kaplan-Meier survival analysis and the log-rank test were used to compare healing times between inpatient and outpatient treatment.

**Results:**

Healing times varied by care setting. The mean time to complete healing was significantly reduced for inpatient care compared to the outpatient setting (median: 34 vs. 60 days; 95% CI 51.2 to 137.8 vs 30.2 to 63.1; p < 0.05). Kaplan-Meier analysis confirmed significantly shorter median healing times in the inpatient group (34 days; 95% CI: 27.2 to 57.4) compared to the outpatient group (69 days; 95% CI: 30.2 to 111.4; log-rank test, *p* = 0.014).

**Conclusion:**

The study highlights the importance of care setting in PU management, with inpatient treatment of PUs grad 2 leading to significantly faster healing than outpatient care.

## Introduction

Pressure ulcers, also known as pressure injuries or decubitus ulcers, are a prevalent and serious complication among individuals with spinal cord injury (SCI) [1]. These injuries usually result from prolonged pressure over bony prominences [2], leading to ischemia, tissue necrosis, and, ultimately, ulceration. Pressure ulcers were classified according to Seiler into four stages based on the depth and extent of tissue damage [3]. Stage 1 is defined as non-blanchable erythema of intact skin. Stage 2 corresponds to partial-thickness skin loss involving the epidermis and parts of the dermis, typically manifesting as an abrasion, blister, or shallow ulcer. Stage 3 represents a defect of the subcutis with involvement of underlying tissues (muscle and connective tissue such as tendons and ligaments) up to the periosteum. Stage 4 includes involvement of the bone with osteomyelitis. Individuals with SCI are particularly susceptible due to impaired mobility, reduced sensation, and altered autonomic regulation, which collectively hinder early detection and timely repositioning [4]. The incidence of pressure ulcers in the SCI population ranges widely, with studies reporting prevalence rates as high as 30–60%, depending on care settings and preventive measures in place. Known risk factors include age, level and extent of intramedullary injury, urinary incontinence, smoking, comorbidities such as diabetes mellitus, cognitive impairment and cardiac disease [5–8]. These factors also play a crucial role in healing of pressure ulcers [9–12].

Beyond the physical consequences, pressure ulcers significantly impair quality of life and pose a substantial economic burden on healthcare systems due to extended hospital stays, surgical interventions, and the need for long-term wound care [13–16]. Moreover, the presence of a pressure ulcer increases the risk of life-threatening infections such as osteomyelitis or sepsis [17, 18].

Despite advances in prevention and management strategies, pressure ulcers remain a persistent challenge in SCI care. The optimal treatment approach is still debated, particularly regarding inpatient versus outpatient management. This is especially true for pressure ulcers grade 2 which can heal with conservative treatment but also progress to grade 3, or even 4.

The objective of this study was to assess the healing progression of PUs grade 2 that were treated conservatively in inpatient versus outpatient settings [19–21].

## Methods

### Study design and patient selection

This study represents a retrospective cohort study. Clinical data were analysed from individuals with SCI resulting in paraplegia (*n* = 22) or tetraplegia (*n* = 15) who received treatment for grade 2 pressure ulcers at the Spinal Cord Injury Centre, University Hospital Balgrist (Switzerland) between July 2017 and July 2019, either in an inpatient or outpatient setting. The study was approved by the Cantonal Ethics Committee Zurich (BASEC-Nr: 2024-00332). The procedure was conducted according to the World Medical Association Declaration of Helsinki and Good Clinical Practice. All patients had given their prior consent for the scientific use and analysis of their data.

Inclusion criteria comprised age over 18 years, a diagnosis of paraplegia or tetraplegia due to spinal cord damage, and the presence of a grade 2 pressure ulcer, classified according to Seiler [3]. Exclusion criteria were the absence of a pressure ulcer or the presence of grade 1, 3, or 4 pressure ulcers (Seiler classification), lack of documented photographic evidence of wound healing, inpatient admission during the healing process in patients otherwise managed on an outpatient basis, or surgical intervention in the wound area. Patients whose wounds had not healed by the time of discharge from the inpatient unit were also excluded. These individuals had been hospitalised for unrelated reasons, and subsequent wound management was carried out in the outpatient setting. The primary endpoints were defined as wound surface area and time to complete healing. Inpatients received daily wound care administered by the nursing staff on the ward, with wound assessments conducted during daily medical rounds. Outpatients were assessed approximately every two weeks by physicians at the outpatient clinic. Home-based wound care was performed either by a home nursing service, the patients themselves, or their family members.

### Data collection

We documented general patient characteristics such as age, gender, level and severity of spinal cord injury, and comorbidities. The level and severity of spinal cord injury were classified according to the ASIA/ISCoS International Standards for Neurological Classification of Spinal Cord Injury (ISNCSCI). Documentation of wound characteristics included localisation, wound size at initial assessment and every follow-up visit, and type of wound dressing.

### Statistical analysis

Descriptive statistics were performed separately for the patients treated in an inpatient and outpatient setting. Values were reported as mean and standard deviation (SD) or median and inter-quartile range. Normality of healing time distributions was assessed using the Shapiro–Wilk test. As normality assumptions were not met, group differences were primarily analysed using the Mann–Whitney U test. Given the a-priori hypothesis that inpatient treatment would be associated with shorter healing times, one-sided tests were applied. Healing time distributions were compared using the Mann–Whitney U test. Additionally, Kaplan–Meier curves and log-rank testing were used to visualise and assess differences in time-to-healing patterns between groups. The analysis reflects a fully observed time-to-event scenario. Therefore, no censoring was necessary. The median healing time was defined as the time point at which the Kaplan–Meier estimated probability of remaining unhealed reached 50%. The log-rank test was used to assess differences in healing times between groups. All tests were one-sided with a significance level of α = 0.05. Data were processed and analysed using custom Python (version 3.10.9) scripts.

## Results

### Patient and wound characteristics

**Fig. 1:**
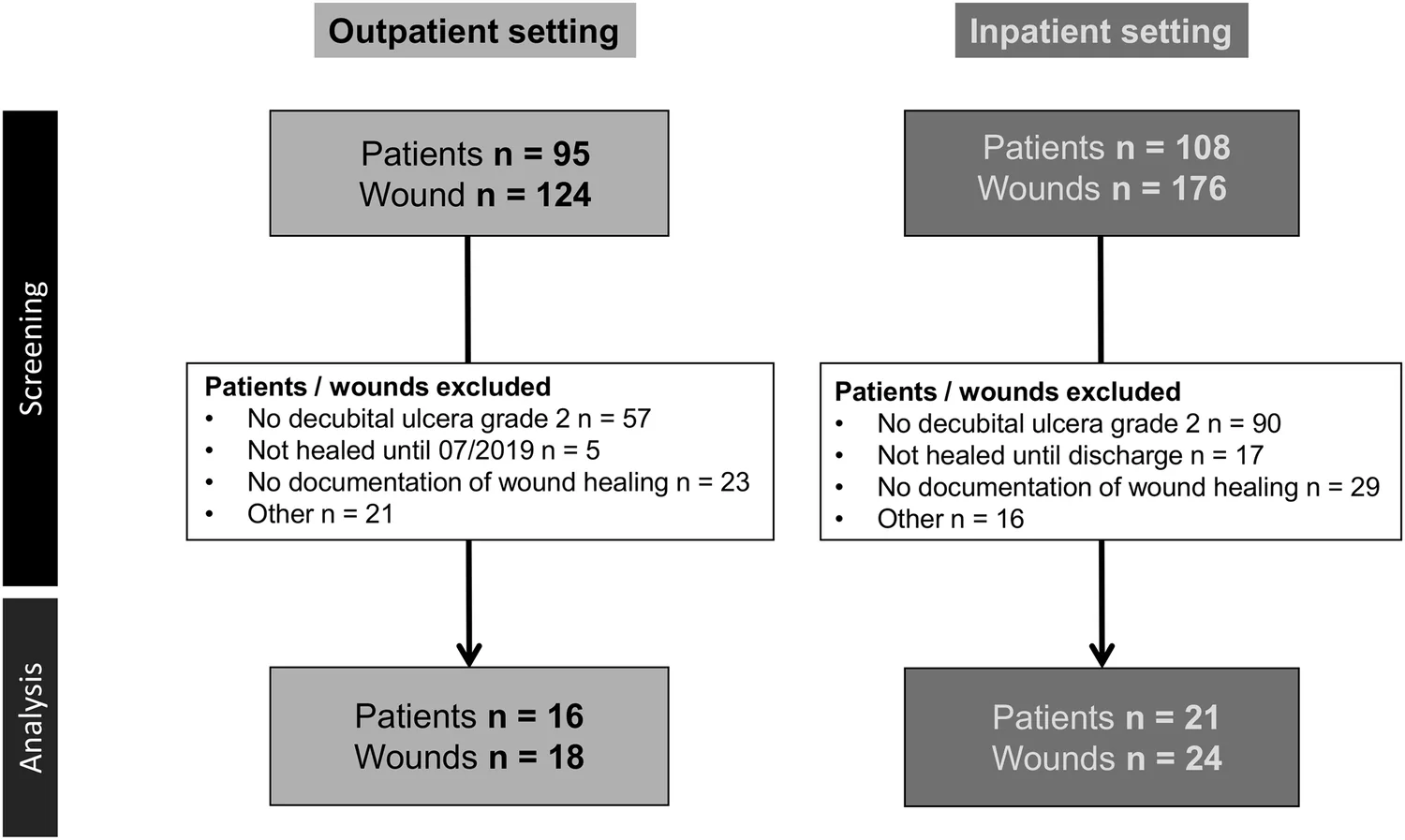
Flow chart of patient selection (07/2017 – 07/2019).

A total of 95 patients with 124 wounds were screened for inclusion in the outpatient group and 108 patients with 176 wounds for the inpatient group. Of these, 16 patients with a total 18 PUs grade 2 remained in the outpatient group, and 21 patients with 24 PUs grade 2 in the inpatient group after applying the inclusion and exclusion criteria (Fig. 1). No missing data were observed for any of the variables included in the analysis.

There were no statistically significant differences between the two groups with respect to age, sex, type and level of spinal cord injury, pressure ulcer location, or type of wound dressing used (Tables 1 and 2). The majority of patients (72%) had pressure ulcers in the gluteal and sacral regions, or in the lower limbs. None of the patients received antibiotic treatment for the PUs.

**Table 1:** Patient Demographics and Clinical Characteristics.

		Outpatient setting	Inpatient setting
Number of patients (n)		16	21
Age in years (mean ± SD)		51.1 ± 18.8	54.4 ± 16.9
	male	13 (81%)	11 (52%)
gender (n)	female	3 (19%)	10 (48%)
Etiology of spinal cord injury (n)	traumatic	10 (62%)	14 (67%)
	inflammatory	3 (19%)	2 (10%)
	malformation	2 (13%)	2 (10%)
	ischemia	1 (6%)	0
	infectious	0	1 (5%)
	tumor	0	2 (10%)
Level of injury (n)	cervical	5 (31%)	11 (52%)
	thoracic	11 (69%)	9 (43%)
	lumbar	0	1 (5%)
Severity of spinal cord damage according to ISNCSCI scale	A	10 (62%)	7 (33%)
	B	1 (6%)	4 (19%)
	C	5 (31%)	5 (24%)
	D	0	5 (24%)
Necessity to use a wheelchair in everyday life (n)		16 (100%)	20 (95%)
Time since injury until occurrence of pressure ulcer in months (median, IQR)		224.5 (94.3–303.8)	144 (33–273)

*n*, number; *SD*, standard deviation, *IQR*, inter-quartile range.

**Table 2:** Wound characteristics and type of treatment.

		Outpatient setting	Inpatient setting
Number of decubital ulcera grade 2 (n)		18	24
localisation (n)	Iliac crest	1	0
	Gluteal region	10	7
	Sacral region	2	8
	Lower leg/ foot/ heel	5	8
	Ear	0	1
Treatment with n (%)	Dexpanthenol (Bepanthen®)	2 (11%)	1 (4%)
	Hydrofiber (Aquacel®, Liquacel®)	14 (77%)	14 (58%)
	Hydrocolloid (Varihesive®, Comfeel)	9 (50%)	9 (38%)
	Foam (PolyMem®, Allevyn®)	3 (17%)	6 (25%)
	Superabsorbent (Zetuvit®)	0 (0%)	1 (4%)
	Lipidocolloid (Urgotyl®, Jelonet®)	0 (0%)	4 (17%)

*n*, number.

### Healing progression

**Fig. 2:**
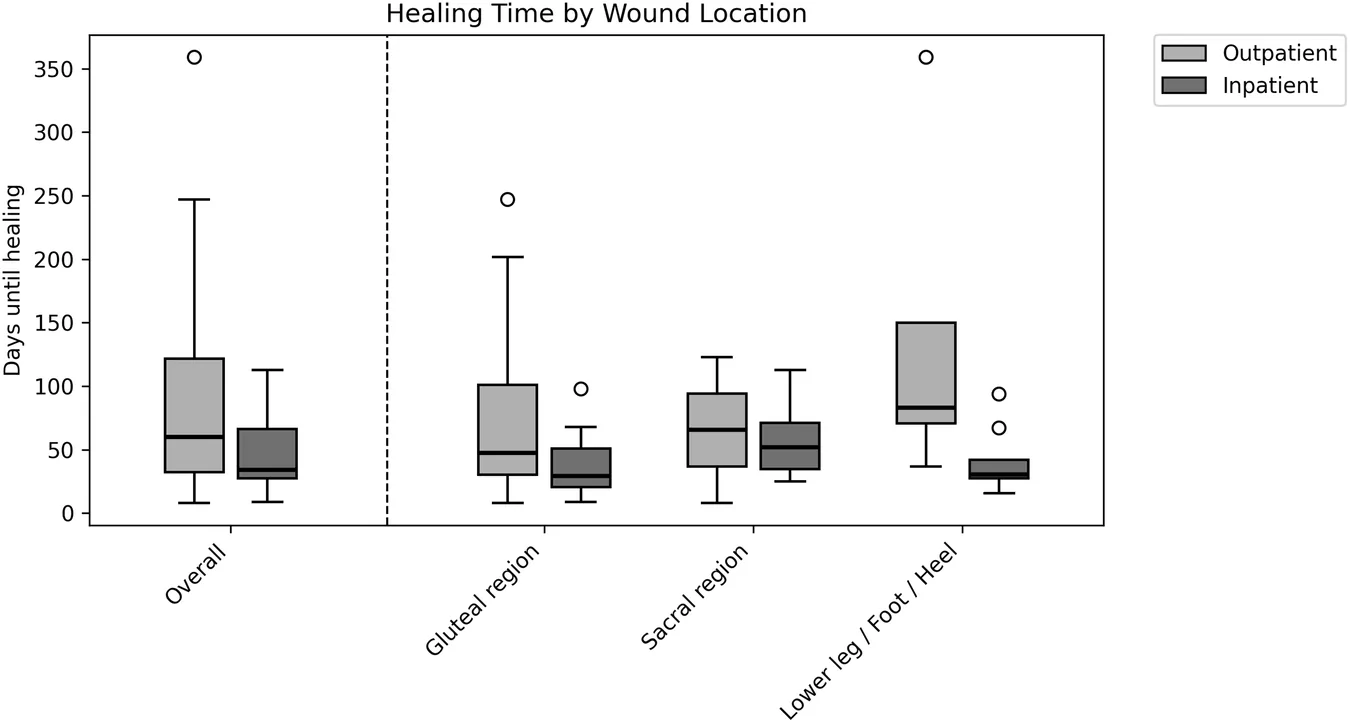
Time to complete healing (outpatient vs. inpatient).

**Fig. 3:**
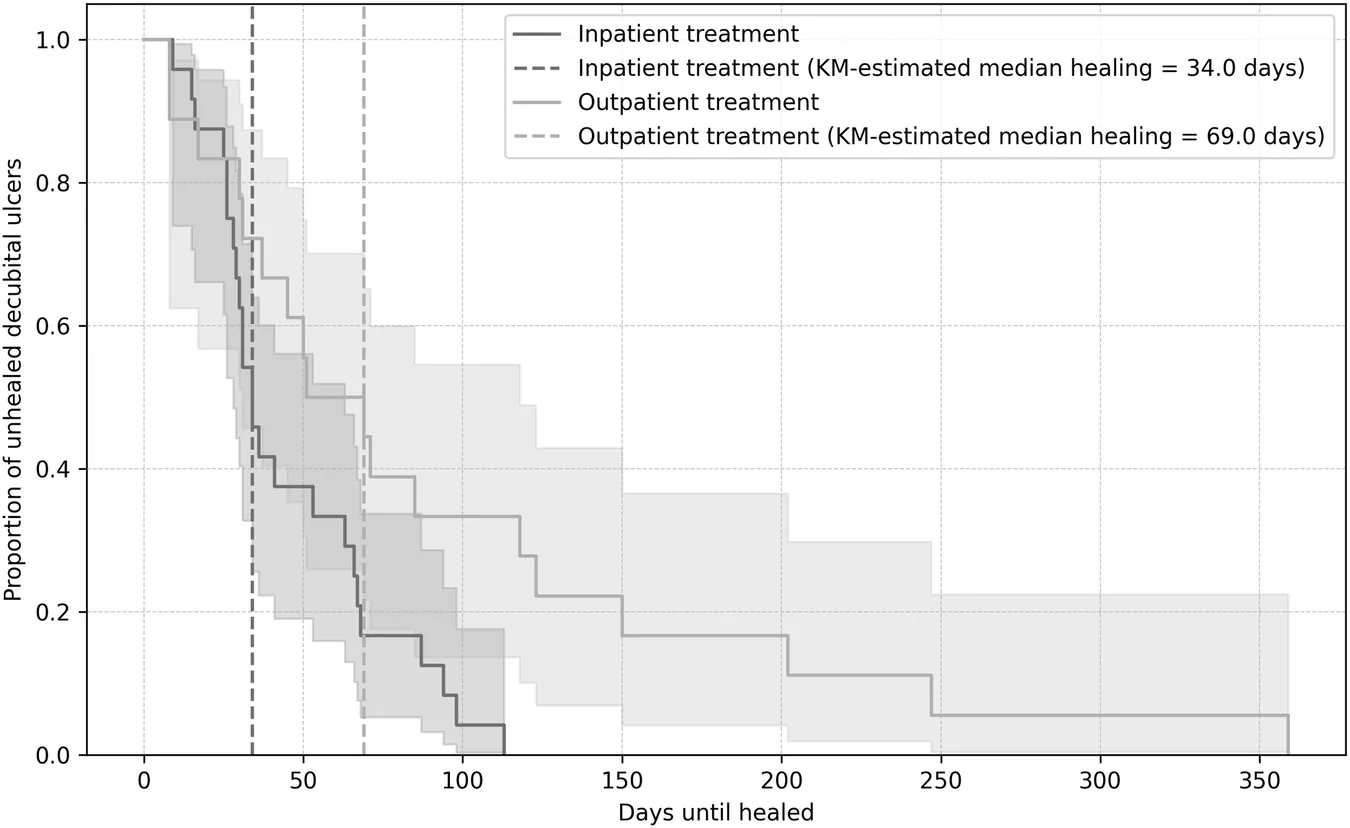
Healing progression (outpatient vs. inpatient).

The duration until complete healing of pressure ulcers was significantly shorter in the inpatient group, being approximately twice as fast compared to the outpatient group (median: 34 vs. 60 days, 95% IQR 27.5 to 66.3 vs 32.5 to 121.8; p < 0.05; Fig. 2). The maximum observed time to complete healing was 113 days in the inpatient group compared to 359 days in the outpatient group (p < 0.05; Fig. 3).

All patients (*n* = 42) achieved complete wound healing during the follow-up period, allowing for uncensored Kaplan-Meier analysis. Median healing time was 34 days (95% CI: 27.2 to 57.4) in the inpatient group and 69 days (95% CI: 30.2 to 111.4) in the outpatient group. The difference was statistically significant (log-rank test, *χ*^2^(1) = 6.023, *p* = 0.014). There was no significant difference in recurrence rate of pressure ulcers between the two groups (5% outpatient, 8% inpatient).

## Discussion

This study compared the healing duration of PU grade 2 in individuals with SCI receiving inpatient versus outpatient setting. Our findings demonstrate that the inpatient group experienced a significantly shorter time to complete healing compared to the patients treated in an outpatient setting, irrespective the localisation of the wound. These results suggest that the inpatient setting may provide a more conducive environment for the effective management and resolution of pressure ulcers in this population.

Several factors may account for the superior outcomes observed in the inpatient group. First, inpatient care typically offers continuous access to multidisciplinary teams, including physicians, wound care specialists, physical therapists, and nutritionists, all of whom play critical roles in pressure ulcer management. This integrated approach allows for timely interventions, close monitoring of wound progression, and rapid adjustments to treatment protocols in response to complications or stagnation in healing.

Second, the controlled environment of inpatient care reduces patient mobility, enforces strict offloading protocols, and ensures adherence to repositioning schedules—all of which are essential components in the management of pressure ulcers [22]. In contrast, patients in outpatient care may face challenges in adhering to these protocols consistently due to limited caregiver support, environmental barriers, or socioeconomic constraints.

Moreover, inpatients are more likely to receive advanced wound care modalities such as surgical debridement, and specialized dressings that may not be as readily available or consistently used in outpatient settings. These advanced interventions are known to accelerate healing and reduce infection rates, which could further explain the improved outcomes in the inpatient group [23, 24].

In this study, 50% of pressure ulcers in the outpatient group healed within 69 days of treatment initiation. Since the prolonged overall healing duration was primarily driven by the nine remaining non-healed ulcers, inpatient admission may be reasonable for patients whose ulcers have not healed within this period. It is also important to consider the potential confounding factors and limitations of our study. While the patient demographics and clinical characteristics did not differ between the two groups, there may be other influential factors that may have affected healing outcomes, such as psychosocial support, patient compliance, or comorbidities. While a multivariate analysis could provide further insights, the sample size in this study is too small to yield reliable results, which is why it was not performed. Due to the retrospective design of this study, no standardised treatment protocol was applied. Wound management, including dressing selection and interventions such as debridement, was performed at the discretion of the treating physician. Additionally, the retrospective nature and single-center scope of the study may limit the generalizability of our findings.

Despite these limitations, the results highlight the potential benefits of inpatient care for individuals with SCI and pressure ulcers. These findings support the need for healthcare systems to evaluate the cost-effectiveness of inpatient versus outpatient strategies, particularly in cases involving advanced or slow-healing wounds. Depending on various factors such as the patient’s occupation with the corresponding duration of incapacity for work, the costs of inpatient care, and the home care situation, the expenses for insurers, and ultimately for society can only be estimated. In an approach not described in detail in this publication, we estimated the costs for outpatient treatment of a grade 2–3 pressure ulcer to be roughly half of those for inpatient treatment in our clinic. Future research should focus on identifying specific elements of inpatient care that most significantly impact healing, and explore whether similar outcomes can be replicated in enhanced outpatient programs or hybrid models of care.

## Conclusion

This study highlights differences in PU healing between inpatient and outpatient settings in individuals with SCI, with inpatient care showing faster recovery. These findings emphasize the importance of care setting in treatment planning, while also underlining the need for tailored treatment strategies. To support evidence-based decision-making and optimise outcomes, further research is necessary to refine PU management protocols in SCI.

## Data Availability

De-identified data from this study are available upon reasonable request by contacting the corresponding author.
